# Advances in patient-derived brain organoid models and emerging ethical questions

**DOI:** 10.1016/j.ebiom.2026.106337

**Published:** 2026-06-10

**Authors:** eBioMedicine

Poised to mitigate many of the barriers associated with biomedical translational research, organoids derived from human tissue often better recapitulate key aspects of human biology than traditional in vitro and in vivo systems, and they might reduce reliance on animal models. Although the first reliably viable organoids took half a century to emerge due to practical challenges in the lab, research using human brain organoids (HBOs) has been growing in number and complexity since the 2010s. By advancing their capacity to model the human brain, researchers are beginning to shed light on disease mechanisms, and personalised therapies are becoming more feasible.

In 2013, a study published in *Nature* modelled microcephaly using human pluripotent stem cell cerebral organoids that self-organised into brain region-like tissue. These models revealed a premature neuronal differentiation mechanism not captured by mouse studies. Now, assembloid platforms, which consist of multiple separately regionalised organoid components, can model more complex tissue–tissue interactions. A four-part human ascending somatosensory assembloid including sensory ganglion-like, dorsal spinal–hindbrain, thalamic, and cortical organoids was shown in a 2025 *Nature* study to functionally assemble the essential components of the human sensory pathway. Researchers also found that treating HBOs with nutrients and signals from astrocytes (*Nature Communications*, 2025) makes neurons mature faster and grow a thicker, more “deep-layer” brain structure. These insights have allowed the development of stronger and more connected electrical activity in culture. The inclusion of microglia-like immune cells in HBOs also helped maturation via cholesterol sharing mechanisms (*Nature*, 2023). Further, a study published in *Cell Stem Cell* in 2025 showed that 3D-printed scaffolds inspired by vasculature can keep midbrain organoids oxygenated and improve recapitulation of drug responses.

Despite these impressive advancements in brain and other organoid research, organoids cannot fully reproduce every functionality and feature, and modelling the brain is inherently challenging. Pitfalls remain, including a lack of high-fidelity cell types, atypical physiology, inefficient vasculature, immature or fatal phenotypes, and substantial remaining variability. Even with these current limitations, HBO research is now advancing remarkably fast, and ethical oversight must keep pace with the research.

Although there is a broad scientific consensus that HBOs currently do not have the circuitry for consciousness or sentience (ie, ability to feel), some technical barriers might be starting to erode. Recent improvements in aspects such as vascularisation and maturation mean that HBOs might become more capable of sustained neural activity. The aim of advances in HBO technology is largely to mimic the brain as closely as possible and, if consciousness or sentience is even remotely possible in the future, strict ethical governance including explicit informed consent guidelines are needed. In May, 2026, the Nuffield Council on Bioethics (NCOB) published proposals to address governance gaps in research involving neural organoids. The report reconfirmed NCOB's stance on the current absence of sentience but warned of a lack of shared understanding of ethical safeguards. NCOB proposed strengthening donor-informed consent and the creation of a central registry. China's 2025 guidelines for organoid research, as well as a recent statement from The International Society for Stem Cell Research, similarly called for careful discussion of these issues. Concerns are shared by the public: in a poll of 326 crowd-sourced respondents in Japan, 36% disapproved of broad consent for HBO research (*Frontiers in Genetics*, 2025). The authors concluded that project-specific consent might be more appropriate. NCOB and others have called for investment in public engagement to explore perspectives from the general population.

An internationally harmonised and dynamic approach to governance is required. A 2025 *European Cell Biology* review highlighted a lack of legal considerations and the need for ethical frameworks. The authors also discussed ethical concerns related to transplantation of human organoids into animals, as well as biocomputing (linking HBOs to computational system or AI), which raises questions about agency and intelligence. Informed consent might require governance-style models whereby donors agree to specific rules for how their cells can be used, which should be upheld on an ongoing basis for all future research.

Despite these challenges, advancing HBO technology from basic research to biomedical applications is on its way to revolutionise diverse fields. Organoids already provide mechanistic insights that were previously obscured and are poised to enable personalised medicine approaches in the years ahead.

